# The Unified Theory of Neurodegeneration Pathogenesis Based on Axon Deamidation

**DOI:** 10.3390/ijms26094143

**Published:** 2025-04-27

**Authors:** Davis Joseph

**Affiliations:** 1 Faculty of Medicine, McGill University, Montreal, QC H3A 0G4, Canada; djoseph@flogen.com; 2 Flogen Technologies Inc., Mount Royal, QC H3P 2T1, Canada

**Keywords:** deamidation, oxidative stress, translational control, neurodegeneration, Alzheimer’s, Parkinson’s

## Abstract

Until now, neurodegenerative diseases like Alzheimer’s and Parkinson’s have been studied separately in biochemistry and therapeutic drug development, and no causal link has ever been established between them. This study has developed a Unified Theory, which establishes that the regulation of axon and dendrite-specific 4E-BP2 deamidation rates controls the occurrence and progression of neurodegenerative diseases. This is based on identifying axon-specific 4E-BP2 deamidation as a universal denominator for the biochemical processes of deamidation, translational control, oxidative stress, and neurodegeneration. This was achieved by conducting a thorough and critical review of 224 scientific publications regarding (a) deamidation, (b) translational control in protein synthesis initiation, (c) neurodegeneration and (d) oxidative stress, and by applying my discovery of the fundamental neurobiological mechanism behind neuron-specific 4E-BP2 deamidation to practical applications in medicine. Based on this newly developed Unified Theory and my critical review of the scientific literature, I also designed three biochemical flowsheets of (1) in-vivo deamidation, (2) protein synthesis initiation and translational control, and (3) 4E-BP2 deamidation as a control system of the four biochemical processes. The Unified Theory of Neurodegeneration Pathogenesis based on axon deamidation, developed in this work, paves the way to controlling the occurrence and progression of neurodegenerative diseases such as Alzheimer’s and Parkinson’s through a unique, neuron-specific regulatory system that is 4E-BP2 deamidation, caused by the proteasome-poor environment in neuronal projections, consisting mainly of axons.

## 1. Introduction

Alzheimer’s and Parkinson’s are serious diseases that cause loss of memory and motor function. The study of these diseases involves several fields of research in biochemical processes such as translational control, oxidative stress, neurodegeneration, and deamidation. Nevertheless, these diseases have been studied separately in basic research and during therapeutic drug discovery.

Here, I provide a short introduction to the key points of each field of research on the above-mentioned biochemical processes, to link them with my discovery in a very recent publication.

Deamidation is a chemical reaction that converts asparagines into aspartates. Asparagine amino acid residues are molecules containing a carbon atom (the α carbon) forming interatomic bonds with three groups of atoms: an amine group (the α-amino group, -NH_3^+^_), a carboxylic acid group (the α-carboxylic acid group, -COO-) and an amide side chain (the R group). Asparagine amino acid residues are converted into aspartyl or isoaspartyl amino acid residues by removing the asparagine’s amide group and transforming it into a carboxylic acid through a deamidation chemical reaction. This reaction disrupts protein function. Flatmark et al. [1] first observed the disruptive nature of deamidation in 1964 in beef heart cytochrome c proteins. Deamidation was first hypothesized to be a biological timer of protein degradation by Robinson et al. [2] in 1970 when they studied peptide models, which are artificially synthesized chains of amino acid residues that form proteins. They established that deamidation was sequence-controlled, meaning that it was controlled by the sequence of amino acid residues surrounding the asparagine or glutamine in the peptide chain. Robinson et al. [2] concluded that asparagine and glutamine amino acid residues were present in various proteins of the living organisms and that their function was to create planned obsolescence of specific proteins to optimize metabolic activity, the chemical activity which maintains life, by causing protein degradation and the replacement of older proteins, no longer of use to the organism, with new ones.

Oxidative stress is the damage caused to cells by reactive oxygen species. Reisz et al. [3] demonstrated that deamidation triggered a recycling mechanism in response to oxidative stress, specifically in erythrocytes, also known as red blood cells. My analysis of the research made by Robinson et al. [2] and Reisz et al. [3] led me to conclude that Reisz et al. [3] unintentionally confirmed Robinson et al.’s [2] work by proving that deamidation causes the replacement of older proteins damaged by oxidative stress with new ones in red blood cells.

Translational control in protein synthesis is the study of protein production control. In 1976, Filipowicz et al. [4] discovered eIF4E, also known as the cap-binding protein (CBP), which paved the way to the creation of the field of translational control research in biochemistry. The eIF4E protein binds to eIF4G, a scaffold protein that allows other proteins to form into groups called complexes. eIF4E alone cannot bind to the 5′ mRNA cap. To do so, it must first bind to eIF4G. eIF4G simultaneously binds to the eIF4A helicase. Together, eIF4E, eIF4G and eIF4A form the eIF4F complex. Haghighat et al. [5] reported that eIF4E, as a subunit of eIF4F, binds to the 5′ mRNA cap, recruiting the ribosome and initiating cap-dependent translation. In 1994, Lin et al. [6] discovered the 4E-binding proteins (4E-BPs). When 4E-BPs attach to eIF4E, eIF4E cannot bind to eIF4G; consequently, the eIF4F complex cannot be formed. This means that eIF4E cannot bind to the 5′ mRNA cap, and protein translation is blocked.

Neurodegeneration is the breakdown of neurons and is involved in memory loss. According to Banko et al. [7], memory loss is caused by a lack of 4E-BP2, the brain’s dominant type of 4E-BP protein. This protein inhibits protein synthesis in the brain by stopping eIF4E from binding to the cap. Bidinosti et al. [8] and Kouloulia et al. [9] showed that neuron-specific deamidation occurs in 4E-BP2 18 days after birth.

In 2023, I discovered [10] that neuron-specific 4E-BP2 deamidation is caused by the proteasome-poor environment in the axons, as postulated in Davis Joseph’s principle [10], which states:

“Due to their proteasome-poor environment, axons increase the protein half-life, which becomes more significant than the deamidation half-life of asparagines, creating neuron-specific deamidation.”

This principle bridges these four fields of research: (1) deamidation, (2) translational control in protein synthesis initiation, (3) neurodegeneration, and (4) oxidative stress, for the following reasons:

1. 4E-BP2 plays a crucial role in memory formation, which is lost during neurodegeneration, as reported by Banko et al [7].
2. 4E-BP2 function is disrupted by deamidation, as shown by Bidinosti et al. [8].
3. 4E-BPs are involved in translational control, as discovered by Lin et al. [6].
4. 4E-BP2 deamidation is implicated in immune responses to oxidative stress, as described by Reisz et al. [3].

In this paper, I will carry out a detailed and thorough critical review of the scientific literature on (1) deamidation, (2) translational control, (3) neurodegeneration, and (4) oxidative stress to achieve the following goals:

(1) Use my discovery [10] on the mechanism behind neuron-specific 4E-BP2 deamidation to link and bridge these four fields.
(2) Develop a Unified Theory, which establishes that the regulation of axon-specific 4E-BP2 deamidation rates controls the occurrence and progression of neurodegenerative diseases.

## 2. Deamidation

In 1964, Flatmark et al. [1] discovered that the main form of cytochrome C in the bovine heart (Cy I) undergoes deamidation and is converted into Cy II and Cy IV. Ow et al. [11] reported that Cytochrome C is a protein implicated in the synthesis of adenosine triphosphate (ATP), which provides energy to cells. Flatmark et al. [12] later confirmed that Cy I is deamidated into Cy II and Cy IV in vitro when exposed to physiological pH, ionic strength, and temperature, and provided conclusive evidence that this process also occurs in vivo using experiments involving radioactive iron. Moreover, Flatmark et al. [12], in 1968, discovered the first turnover rate of a mammalian protein converting into its deamidated form. Then, in 1974, Robinson et al. [2] measured the deamidation half times (half-lives) of cytochrome c in pentapeptide models, which are artificially created peptides, and demonstrated that deamidation was a nonenzymatic, sequence-controlled event. Their results confirmed that deamidation initiates cytochrome c degradation.

My critical analysis of the results mentioned above made me conclude that asparagines and glutamines, prone to deamidation, are essential elements of dysfunction inserted into cytochrome c that modify the function of proteins at a specific time.

In 1970, Lai et al. [13] discovered that deamidation was also found in the aldolase protein, which, as described by Du et al. [14], is also involved in ATP synthesis. Lai et al. [13] found that the asparagine (Asn) of the COOH-terminal sequence in the α subunit of aldolase was substituted for an aspartate (Asp) amino acid residue in the β subunit and suggested this change was caused by deamidation. Midelfort et al. [15] later confirmed that the conversion from the α subunit to the β subunit was caused by deamidation. Midelfort et al. [15] also found that the deamidation half-time in aldolase was 8 days and equal to the aldolase protein’s half-life. In 1974, McKerrow et al. [16], in the Robinson lab, found that the Gly-Ser-Asn-His-Gly peptide, which contained the four c-terminal amino acid residues found in aldolase, had a deamidation half-life of 6.4 days +/− 0.5, which was consistent with the 8-day timespan of in vivo aldolase deamidation half-life and aldolase protein half-life as discovered by Midelfort et al. [15].

My analysis shows that the key finding in McKerrow et al. [16] was that Aldolase deamidation was spontaneous (nonenzymatic) and determined by the peptide sequence.

An essential observation in my analysis of the publications mentioned above is that Robinson et al.’s [2] conclusion that deamidation causes protein degradation, which he refers to as “programmed obsolescence”, contradicts the experimental findings of Lai et al. [13] and Morse et al. [17]. Lai et al. [13] found that deamidation in aldolase had the opposite effect of what deamidation had in cytochrome c. Indeed, the aspartyl-histidine bond created from deamidation is very resistant to carboxypeptidase digestion, which is the form of protein degradation undergone by aldolase. Morse et al. [17] also found that the β subunit, later found to be the deamidated subunit, was more resistant to degradation than the α subunit. Midelfort et al. [15] also assumed that the deamidated aldolase subunit degraded at an equal rate to the undeamidated α subunit, because both subunits have identical amino acid residue sequences except for one asparagine located at the surface of the protein. However, as per Lai et al. [13] and Morse et al. [17], this single asparagine residue significantly decreased resistance to degradation.

Based on this information, I found that the experimental evidence strongly suggests that deamidation disrupts protein function but does not necessarily cause protein “programmed obsolescence” as postulated by Robinson et al. [2]. Moreover, I state that the deamidation of one asparagine can significantly alter protein degradation rates despite Midelfort et al.’s [15] assumptions.

Arthur Robinson’s work managed to conclude that primary peptide sequence, as well as secondary and tertiary protein structure, were the primary determinants of deamidation half-life in both asparagine residues and glutamine residues (hydrogen bonding was later found to also play a significant role in deamidation half-life). Arthur Robinson’s work continued into the study of deamidation in histones. These proteins together form nucleosome core particles that DNA wraps around to form chromosomes, as described by Martire et al. [18]. Robinson et al. [19] observed significant deamidation in in vitro peptide sequences found in histones and concluded that histone deamidation must alter chromosomal function by modifying nucleosome structure. Subsequently, Lindner et al. [20] found that deamidation occurs in histones in vivo. Lindner et al.’s [20] findings suggested that age-dependent deamidation directly affects histone-DNA interactions due to deamidation’s ability to reduce the positive charge on the N-terminal domain of histones. Lindner et al. [20] concluded that by modifying histone-DNA interactions, deamidation may be the leading cause behind an age-dependent decrease in transcription (the production of RNA) in the brain and other tissues, because deamidation may cause changes in chromosome structure. Medvedev et al. [21] found that changes in chromosome structure (chromatin conformation) caused this decrease in transcription.

Solstad et al. [22] also performed similar experiments on human phenylalanine hydroxylase (hPAH), a protein which, as explained by Tomé et al. [23], adds a hydroxyl group (-OH) to the L-isomer of the phenylalanine amino acid residue and converts it into an L-tyrosine amino acid residue. Solstad et al. [22] discovered that deamidation of the hPAH protein creates multiple molecular forms of this protein, a concept known as microheterogeneity. As proof of deamidation occurring in hPAH and causing microheterogeneity, Solstad et al. [22] showed that ammonia (NH_3_) was released when hPAH converted from one form to another, and this is consistent with what happens during deamidation. Moreover, Solstad et al. [22] found more evidence of deamidation when looking at the differences in isoelectric points (pI) between each form, the pI being the pH at which the molecule has a neutral charge. Solstad et al. [22] found that the differences in pI between each form of hPAH were consistent with the differences in pI that would occur if one, two, and three asparagine residues of hPAH were consecutively substituted for aspartates. Other important findings for this protein by Solstad et al. [22] were that deamidation (1) significantly increased the protein’s catalytic efficiency, meaning that hPAH generated more products at a faster rate after deamidation and (2) significantly increased hPAH’s susceptibility to partial substrate inhibition when exposed to higher, nonphysiological concentrations of phenylalanine. Solstad et al. [22] also found that, after deamidation, hPAH was more susceptible to tryptic proteolysis.

My conclusion on Solstad et al.’s [22] research on deamidation in hPAH is that Solstad et al. [22] gave further proof that protein function alteration by deamidation can be beneficial to the protein, because it increased the protein’s catalytic efficiency in hPAH.

Takemoto et al. [24] found that longer-lived proteins like the γ-S crystallin protein located in the lenses of the human eyes were highly resistant to deamidation. Takemoto et al. [24] found no detectable deamidation in asparagine 143 of the VLEGVWIFYELPNYR amino-acid residue sequence in the crystalline protein.

To understand the reasons behind γ-S crystallin’s high resistance to deamidation, I analyzed the organic chemistry behind what renders an asparagine in a peptide susceptible to deamidation in vivo. The goal was to conceive a new in vivo deamidation biochemistry flowsheet that will be given in this article. This analysis is shown below.

I first identified the initiation factors behind deamidation. Kato et al. [25] described that deamidation initiation depends entirely on the likelihood that the main chain amide nitrogen of the C-terminal amino acid (N + 1) will donate its electrons to the asparagine amide carbon, a concept defined as nucleophilicity. Yokoyama et al. [26], Michael Bidinosti [27], and Vlasak et al.’s [28] works describe the nucleophilic attack by the main chain amide nitrogen as the initiating step of the deamidation reaction, a process known as cyclization. This nucleophilic attack is theoretically correct, and intramolecular reactions generally favor nucleophilic attack due to increased electrophile-nucleophile proximity. However, based on the principles of electron delocalization and resonance described in D. R. Klein’s book [29], my analysis shows that the amide nitrogen is a weak nucleophile because its resonance with the carbonyl amide stabilizes the molecule by delocalizing nitrogen lone pair electrons onto the carbon-nitrogen bond. This reduces electron-electron repulsion and prevents significant nucleophilic attack. Therefore, nucleophilic attack from the amide is improbable. However, after I analyzed paper after paper, I found that main chain amide nucleophilic attack when describing in vivo deamidation is a common mistake propagated in the scientific literature for decades. Instead, I inferred that spontaneous deamidation and asparagine cyclization require main chain nitrogen deprotonation by the hydroxyl ion (OH-) or another amino-acid residue for the deamidation reaction to progress and that direct nucleophilic attack from the deprotonated main chain amide nitrogen to the carbonyl carbon of the asparagine is the only plausible initiation step in vivo. Proof of the necessity for N + 1 main chain nitrogen deprotonation to occur during deamidation is found in Takahashi et al. [30], who showed that deamidation has an exceptionally short half-time when flanked at the C-terminal by the histidine amino acid residue because histidine deprotonates the main chain amide nitrogen. I postulate that this short deamidation half-time in the case of asparagine neighboring histidine is unique, because, according to Robinson et al.’s data [31], when large amino acid residues similar in size to histidine, like tyrosine, flank asparagines at the c-terminal, deamidation has a significantly longer half-time.

I reason that only the increased deprotonation of the main chain nitrogen allows for short deamidation half-times when histidine is present at the c-terminal, demonstrating the importance of main chain nitrogen deprotonation in deamidation.

However, as per my analysis, the main chain nitrogen deprotonation is not the only factor determining asparagine’s susceptibility to deamidation. Again, Robinson et al. [31] describe that glycine, the smallest amino acid residue, gives asparagine its shortest deamidation half-time, even though glycine plays no role in deprotonation.

My analysis led me to conclude that another important factor is at play when determining deamidation rates. Based on my review of the scientific literature, I determined that this factor is the molecule’s orientation, which is caused by the repulsion of electron clouds between atoms depending on the size and orientation of the other molecules surrounding the asparagine, a concept defined as steric hindrance by D.R. Klein [29]. Besides main chain nitrogen deprotonation, I deduce that deamidation mainly depends on the conformation (structure) and orientation of the asparagine and the C-terminal amino acid flanking it. As described by Capasso et al. [32,33] and Clarke [34], the three-dimensional positions of the atoms of the asparagine residues must have tetrahedral angles of −120 degrees and +120 degrees for ψ and χ angles, respectively, for the asparagine to undergo deamidation. This positioning brings the main chain amide nitrogen and the γ carbonyl carbon (the carbon to be attacked by the nitrogen) to their closest possible proximity, 1.89 Angstrom. If the dihedral angles deviate from these values, say ψ and χ values of +60 and −60 degrees, respectively, the γ carbonyl carbon and the main chain nitrogen will be too far away from each other, at a distance of 4.89 Angstrom. According to Clarke [34], at this distance, deamidation will not be possible. As per Capasso et al. [32], the dihedral angles that favor deamidation, however, create a very high energy conformation in the Ramachandran plot and, therefore, cause instability.

Based on my analysis, this must be due to steric hindrance. This makes the desired conformation for deamidation highly unlikely to occur unless the asparaginyl residue has unrestricted flexibility. Based on my analysis, this is another reason why bulky C-terminal amino acids disfavor deamidation and glycine favors it: the former restricts flexibility due to steric hindrance, and the latter increases flexibility due to the absence of steric hindrance, because of glycine’s small size. This also explains why deamidation half-lives are longer in structured proteins compared to primary peptides synthesized with identical sequences, as seen in Capasso et al. [32] and Robinson et al.’s works [19]. Based on my investigation of the scientific literature, the rigidity of protein structure restricts flexibility. The importance of three-dimensional positioning in deamidation also explains the role of hydrogen bonding in deamidation half-life, because, as seen in Capasso et al. [32], hydrogen bonding is known to change the conformation of molecules.

In summary, based on my critical literature review, deamidation initiation is entirely dependent on two factors:

(1) Access to the solvent for main chain nitrogen deprotonation
(2) Flexibility of the asparaginyl residue

The importance of these two factors is clearly demonstrated in asparagine 143 of γ-S crystallin. As stated above, Dr. Takemoto’s work [24] provided conclusive evidence that deamidation at Asn 143 does not occur when found in γ-S crystallin. This is because neither of the deamidation initiation factors I identified above is present. Asn 143 has minimal flexibility inside the γ-S crystallin protein structure and has no exposure to the solvent. However, according to Lapko et al.’s work [35], in cataract lenses where γ-S crystallin misfolds and aggregates, Asn 143 is exposed to the protein’s surface, and deamidation in Asn 143 is significantly observed.

My critical analysis of the literature leads me to conclude that this phenomenon occurring in cataract lenses confirms the importance of the two deamidation initiation factors I deciphered above, because:

(1) Increased protein misfolding causes protein structure to break down, allowing for increased flexibility in peptide chains
(2) Increased exposure to the solvent increases the susceptibility of main chain nitrogen deprotonation from hydroxyl groups found in the solvent.

These two factors led to significant deamidation in Asn 143, previously undetectable in structured crystallin proteins, as seen in the evidence provided by Takemoto et al. [24] and Lapko et al. [35].

Moreover, Takahashi et al. [30], Kirikoshi et al. [36], and Kato et al. [25] have shown that the subsequent cyclization steps of deamidation, after deprotonated main chain amide nucleophilic attack, involve a gem-hydroxylamine intermediary that undergoes nitrogen protonation and subsequent ammonia expulsion in vivo. The existence of this molecule is in contradiction with a cyclization process proposed by Dunkelberger et al. [37] involving azanide expulsion following nucleophilic attack (note: azanide expulsion is presented incorrectly as a one-step process by Dunkelberger et al.; it is a two-step process as shown in my paper [10]). Again, both mechanisms are theoretically correct, but my investigation aims to identify the prevalent mechanism in vivo.

Based on my critical literature review, azanide expulsion is favored under highly basic conditions, based on amide hydrolysis mechanisms described in D.R. Klein’s work [29]. However, my analysis of Slyvka et al.’s [38] characterization of the azanide molecule shows that it is a highly reactive molecule not found in vivo. Therefore, I infer that gem-hydroxylamine formation is favored over azanide expulsion in vivo. Based on Takahashi et al.’s work [30], succinimide is formed after amine protonation and subsequent ammonia expulsion from gem-hydroxylamine. My analysis shows that succinimide is a highly stable intermediary due to multiple lone electron pair delocalizations by resonance, and this is based on the fundamental principles of organic chemistry described by D.R. Klein [29]. I deduce that succinimide formation is the driving factor behind aspartate formation in deamidation due to succinimide’s high stability as a molecule.

After succinimide formation, there are two ways that hydrolysis of succinimide can occur because both nitrogen-carbonyl bonds are susceptible to hydroxyl nucleophilic attack. Succinimide hydrolysis can form two products: (1) aspartate or (2) isoaspartate. These products are formed in a roughly 1:3 ratio, respectively, as demonstrated by Geiger et al. [39]. However, as shown by Böhme et al. [40] and Murray et al. [41], because isoaspartate tends to disrupt the function of proteins and enzymes, L-isoaspartyl methyltransferase (PIMT) converts isoaspartates to aspartate.

Altogether, all these above-mentioned works on the mechanistic pathway of deamidation led me to conclude that deamidation cannot be completed in vivo without basic and acid catalysis and proper three-dimensional positioning of the asparagine amino acid residue.

**Figure 1:**
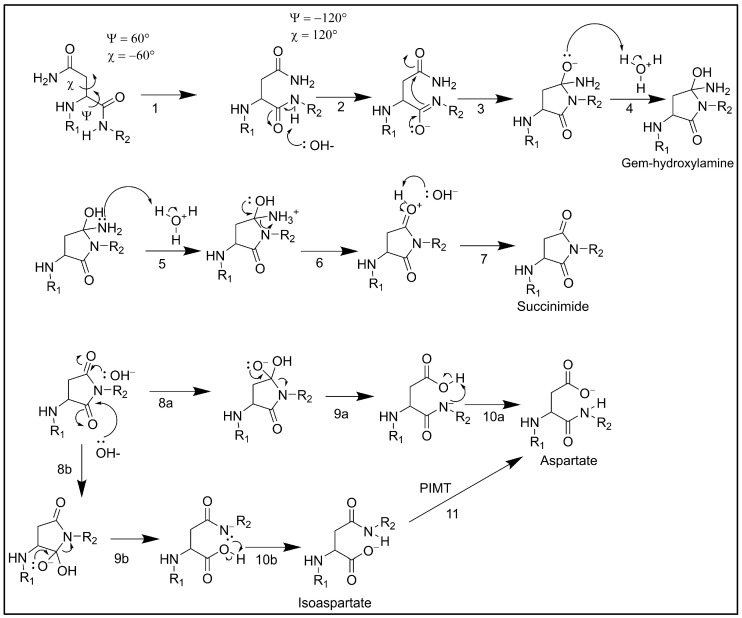
New biochemical flowsheet of in vivo deamidation based on subatomic electron delocalization.

In my previous paper [10], I showed an organic chemistry flow sheet to help the reader understand the oxidative nature of deamidation. After careful analysis and critique of all literature on the organic chemistry behind deamidation, as seen above, I managed to invent a new biochemical flowsheet specifically for in vivo deamidation. This is given in Figure 1. This figure synthesizes all relevant information from the scientific literature regarding the in vivo deamidation reaction into one biochemical flow sheet.

Step 1 signifies the conformational change required for the molecule to achieve the dihedral angles favorable for deamidation. Step 2 is the deprotonation step of the main chain nitrogen atom at the C-terminal. Step 3 involves nucleophilic attack of the main chain nitrogen towards the γ carbon of the asparaginyl carbonyl. Step 4 is the protonation of the asparaginyl oxygen, leading to a gem-hydroxylamine molecule. Step 5 is the protonation of the amine. Step 6 involves deammoniation, the loss of an ammonia group. Step 7 involves deprotonating the oxygen molecule to form the succinimide molecule. The succinimide molecule is a highly stable intermediary due to multiple resonance forms. Step 8a involves a hydroxyl nucleophilic attack that leads to an aspartyl side-chain, whereas step 8b involves a hydroxyl attack that leads to isoaspartyl formation. Steps 9a and 9b both involve decyclization. Steps 10a and 10b both involve deprotonation, leading to the formation of aspartate and isoaspartate, respectively. Step 11 involves the conversion of isoaspartate to aspartate via the PIMT protein.

My crucial contribution is my demonstration of electron delocalization throughout the deamidation reaction. This allows the reader to fundamentally understand every step of the deamidation reaction at the subatomic level.

Deamidation is also present in many other proteins.

Emily et al. [42] described that BH3 domain-only proteins activate Bak and Bax proteins, which promote mitochondrial cytochrome C release. Gross et al. [43] described that this event triggers the activation of cell death-inducing caspase proteins, which trigger apoptosis (cell death). Deverman et al. [44] discovered that DNA-damaging antineoplastic agents induce deamidation in two asparagines of the unstructured loop in the B-cell lymphoma 2 (Bcl-2) protein Bcl-XL. This protein inhibits the proapoptotic activity of BH3 domain-only proteins. Deverman et al. [44] explain that deamidation disrupts and inactivates Bcl-XL, allowing proapoptotic activity to progress. They also state that Bcl-XL deamidation induces the apoptosis of tumor cells. Deverman et al. [44] explain that the retinoblastoma tumor suppressor protein (Rb) suppresses Bcl-XL deamidation in p53 null mouse embryo fibroblasts (tumor cells generally lacking p53 activity), and this prevents cisplatin-induced apoptosis.

My conclusion, based on this research, is that deamidation inactivation leads to chemotherapy-resistant cancer cells.

Sun et al. [45] also found that deamidation occurs in the triosephosphate isomerase protein at Asn 71 and Asn 15, causing major structural changes and degradation of the protein. Interestingly enough, Sun et al. [45] state that deamidation of Asn 71 was found to be a prerequisite for the deamidation of Asn 15.

This led me to conclude that one deamidated amino-acid residue could lead to a cascade of subsequent deamidation reactions.

Capasso et al. [32] also found that deamidation occurs in ribonuclease’s Asn 67 and Asn 3 as well as in peptides with similar sequences. However, they state [32] that the reaction was found to be much slower in the protein compared to the in vitro peptide sequence.

I infer that this is most likely because of steric hindrance and the restricted flexibility of the amino acid residues inside the protein structure.

Shimizu et al. [46] and Robinson et al. [47] discovered that deamidation is also found in amyloid β polypeptides and α-synuclein, respectively. These two proteins are characteristic of Alzheimer’s and Parkinson’s disease, respectively, and will be discussed in detail in the subsequent sections below.

Using the solid phase peptide synthesis technique invented by Merryfield et al. [48], Robinson et al. [31] synthesized what they believed were the 800 possible amino acid sequences involving asparagine deamidation.

## 3. Translational Control

The field of translational control had its beginnings in 1976 when Filipowicz et al. [4] discovered the eIF4E protein and its causal link to translation initiation. Two years later, in 1978, Sonenberg et al. [49] confirmed Filipowicz et al.’s findings by repeating exactly the same experiments and adding a crosslinking chemical to increase the stability of the mRNA-protein complex.

In 1983, Vanessa Majo [50] discovered that eIF4E is an oncogene. 7 years later, Lazaris-Karatzas et al. [51] confirmed her findings.

I set out to analyze and synthesize all relevant scientific literature on protein translation initiation and translational control, to create a new, exhaustive flowsheet of translational control in protein synthesis initiation.

I first started by looking at the scientific data relating to translational control. As described by Lamphear et al. [52], eIF4G is a scaffold protein that binds to eIF4E before eIF4E binds to the 5′ mRNA cap. Haghighat et al. [5] found that eIF4G binding to eIF4E has been shown to be necessary for efficient eIF4E binding to the 5′ cap and that the absence of eIF4G binding to eIF4E leads to very inefficient cap binding. As described by Ray et al. [53] and Sachs et al. [54], eIF4G also simultaneously binds to eIF4A, which is a helicase that unwinds the 5′ untranslated region (UTR) of mRNA and facilitates ribosome binding to the mRNA. As discovered by Rogers et al. [55], eIF4B and eIF4H increase the efficiency of eIF4A helicase. As described by Cencic et al. [56], the eIF4A helicase has been found to be a potent drug target to stem the progression of metastases, and drugs such as MG-002 have been shown to inhibit tumor growth. As described by Richter et al. [57], the eIF3 protein is bound to the 40S ribosomal subunit as part of the 43S protein translation preinitiation complex and recruits this complex to the mRNA by also binding to eIF4G.

As Haghighat et al. [5] explain, one hypothesis based on Joshi et al. [58] assumed that eIF4E binds to the 5′ mRNA cap first and binds to eIF4G afterwards because Joshi et al. [58] found that eIF4G sometimes complexed with ribosomal preinitiation complexes instead of eIF4E. However, this hypothesis was disproven by Lee et al. [59] and Pelletier et al. [60], because they showed that the separation of eIF4G from eIF4E inhibited cap binding by eIF4E. Moreover, as Grifo et al. [61] described, eIF4B could not bind efficiently to the mRNA without the presence of the eIF4F complex.

Based on my analysis, these three findings validated the hypothesis of previous scientific literature that eIF4E cannot bind to the cap if it is not part of the eIF4F complex.

The gene of the 4EHP protein (EIF4E2), which is an eIF4E-like protein, was discovered and characterized by Gao et al. [62] and later by Mao et al. [63]. Rom et al. [64] cloned and characterized the 4EHP protein. Christie et al. [65] explain that the 4EHP protein is 30% identical and 60–65% similar to eIF4E and, like eIF4E, 4EHP can bind to the 5′ cap. However, Christie et al. [65] state that 4EHP cannot interact with eIF4G and, therefore, 4EHP represses translation when binding to the cap.

Lin et al. [6] discovered that 4E-BPs are proteins that inhibit translation by binding to eIF4E. Subsequently, Azpiazu et al. [66] demonstrated that 4E-BP phosphorylation dissociates 4E-BPs from eIF4E. Mader et al. [67] also showed that 4E-BPs compete with eIF4G for binding to eIF4E because eIF4E binds to an amino acid residue sequence motif contained in both 4E-BPs and eIF4G. These motifs are the amino acid sequences that connect proteins. Kimball et al. [68] demonstrated that insulin increased 4E-BP phosphorylation and, consequently, significantly decreased 4E-BP-eIF4E interaction and increased protein translation. As further proof of insulin’s role in 4E-BP-eIF4E interaction, Jefferson et al. [69] discovered that insulin deficiency in diabetes increased 4E-BP-eIF4E interaction 3-fold.

Altogether, the above-mentioned findings by researchers affiliated with the John C. Lawrence Jr. laboratory uncovered the fundamental link between translational control and diabetes.

Nusbaum et al. [70] discovered that a gene encoded a protein named C8ORF88. 17 years later, this protein was characterized by Pugsley et al. [71]. Pugsley et al. [71] state that this protein is found mainly in spermatids, the immature precursors of spermatozoa, and is a 4E-BP protein which inhibits eIF4E, but is not regulated by phosphorylation.

Minich et al. [72] discovered that eIF4E phosphorylation increases eIF4E’s affinity to the cap. Li et al. [73] discovered that phospho-eIF4E is implicated in the accumulation of pathological hyperphosphorylated tau protein in the Alzheimer’s brain, a disease which I will study in detail in the next section.

Banko et al. [7] and Gkokas et al. [74] also discovered that 4E-BP2 in particular is crucial to memory formation and social behavior. 4E-BP2 is the dominant paralog (type) of 4E-BP proteins in the brain. Bidinosti et al. [8] and Kouloulia et al. [9] found that 4E-BP2 undergoes deamidation 18 days after birth. Kouloulia et al. also describe that deamidation overexpression leads to a decrease in the activity of proteins related to cortical development, mitochondrial activity, as well as nuclear factor κ-light-chain-enhancer of activated B cells (NF-κB) activity.

This may seem counterintuitive, since 4E-BP2 deamidation prohibits 4E-BP2 from inhibiting eIF4E. Based on the evidence, I hypothesize that this may be because proteins previously inhibited by 4E-BP2 are overexpressed after 4E-BP2 deamidation, and the resources of the cell now predominantly shift to these proteins, to the detriment of others. I also hypothesize that this shift in protein inhibition may also have something to do with the increased affinity of deamidated 4E-BP2 for the raptor component of mTORC1, which has been shown, as demonstrated by Kouloulia et al., to reorient mTORC1 activity towards proteasomal degradation activity instead of phosphorylation activity.

The fragile X mental retardation protein (FMRP) and the cytoplasmic FMR1-interacting proteins (CYFIP1 and CYFIP 2) were found to play a crucial role in translational control in the brain. Verkerk et al. [75] first discovered the FMR1 gene. Afterwards, Siomi et al. [76] characterized the function of the FMRP protein encoded in the FMR1 gene. Darnell et al. [77], Ascano et al. [78], Maurin et al. [79], Li et al. [80] and Richter et al. [81] all played an important part in describing how the fragile X mental retardation protein (FMRP) was found to bind to mRNA in the central nervous system and inhibit protein translation as well as its other functions. Schenck et al. [82] identified the CYFIP protein family. In their work, Schenck et al. [82] describe that FMRP binds to the FMR1-interacting proteins CYFIP1 and CYFIP2, both 4E-BP proteins. Napoli et al. [83] describe how FMRP has been found to inhibit translation through CYFIP1’s interaction with eIF4E. Napoli et al. [83] also state that CYFIP2 is thought to have the same regulatory function as CYFIP1, because they share the same binding motif. Clifton et al. [84] describe how FMRP is predominantly expressed in synapses (where pre-synaptic axon terminals connect to post-synaptic dendrites), both in the pre-synapse (axon terminal) and the post-synapse (dendrites). Clifton et al. [84] and Davenport et al. [85] also described how CYFIP1 and CYFIP2 are predominantly expressed in the synapses of excitatory and inhibitory neurons. Kim et al. [86] and Hsiao et al. [87] both describe how CYFIP1 and CYFIP2 have been found to regulate the development and function of neurons, both in the presynapse and the postsynapse of hippocampal neurons. Interestingly, Tiwari et al. [88] also describe how CYFIP 1 and 2 are multifunctional proteins. Tiwari et al. [88] state that CYFIP1 and CYFIP2 are part of the Wiskott-Aldrich syndrome protein family verprolin-homologous protein complex;WAVE for short. De Rubeis et al. [89] describe how the brain-derived neurotrophic factor (BDNF) induces CYFIP1 to convert from a globular conformation to a planar conformation, and that the Ras-related C3 botulinum toxin substrate 1 (Rac1) protein promotes this process. Chen et al. [90] describe how CYFIP1 in a planar conformation can no longer interact with eIF4E and is instead recruited to the WAVE regulatory complex (WRC). De Rubeis et al. [89] explain how, when CYFIP1 is recruited to the WRC complex and dissociated from eIF4E, CYFIP1 interacts with the NCK-associated protein 1 (NCKAP1) and promotes actin polymerization and protein translation.

Translational control has also been linked to autism spectrum disorder (ASD) and Schizophrenia. English et al. [91] describe how post-mortem Alzheimer’s brains have been found to have reduced translation at the soma, the neuron cell body, which leads to thinning of the soma and a reduction in total brain mass. Based on this scientific literature, I postulate that this is most likely due to reduced eIF2 protein in the schizophrenic brain. In fact, Kimball et al. [92] and Pain et al. [93] describe how eIF2 plays a crucial role in translation initiation. For translation to commence, the initiator Met-tRNA must bind to eIF2 and guanosine tryphosphate (GTP), a molecule that provides the necessary energy for translation initiation. Together, they form the ternary complex and associate with the 40S ribosomal subunit. After the association between 40S and the ternary complex is established, the 43S preinitiation complex is formed. The 43S preinitiation complex, as mentioned above, is recruited to the 5′ cap of the mRNA-eIF4F complex thanks to binding between eIF3 and eIF4G to create the 48S preinitiation complex and initiate translation. Passmore et al. [94] describe how eIF1 and eIF1A together open the mRNA binding channel by attaching to the 40S ribosomal subunit, together with eIF3, prior to 40S association with the ternary complex. Pain et al. [93] also explain that eIF3 and eIF1A binding to 40S delays 40S reassociation with the 60S subunit long enough to allow for 43S preinitiation complex formation and subsequent protein synthesis. Later, Pain et al. [93] state that after binding to the mRNA, the 48S preinitiation complex will detect the AUG initiation codon. According to Unbehaun et al. [95] and Pisarev et al. [96], once 48S detects the AUG codon, eIF5 binds to eIF2 and triggers the hydrolysis of its GTP. Both these groups of authors also state that, subsequently, the eIF2-GDP complex is detached from the 48S preinitiation complex by eIF5B. The 40S subunit subsequently binds to the 60S ribosomal subunit, forming the 80S ribosome and allowing protein translation to commence. Pain et al. [93] continue to explain that the GDP resulting from hydrolysis is exchanged for GTP via eIF2B, also known as a guanine nucleotide exchange factor (GEF), allowing translation to continue. However, Kimball et al. [92] explain that, when eIF2 is phosphorylated at serine 51 in its alpha subunit, eIF2’s affinity for eIF2B increases, inhibiting eIF2B activity. As a consequence, eIF2 can no longer recycle its GTP, and translation comes to a halt. Kimball et al. [92] described that this process is used as a defense mechanism by cells to avoid producing viral proteins, but English et al. [91] explain that a reduction of eIF2 activity in neurons drives, in part, the pathogenesis of schizophrenia. Pathania et al. [97] and Haddon et al. [98] explained that deletion of the CYFIP1 coding gene is also responsible for a two-to-four-fold increase in the risk of Schizophrenia, autism, and intellectual disability.

Based on my analysis, this increase in neuronal dysfunction is most likely because of the alteration of the transcriptome associated with local synaptic translation. This is confirmed by Clifton et al. [99], who have demonstrated that the alteration of the transcriptome associated with local synaptic translation has been found to be strongly linked to the progression of schizophrenia and intellectual disability.

Pathologies linked to the central nervous system and neurodegeneration will be discussed in detail in the next section below.

**Figure 2:**
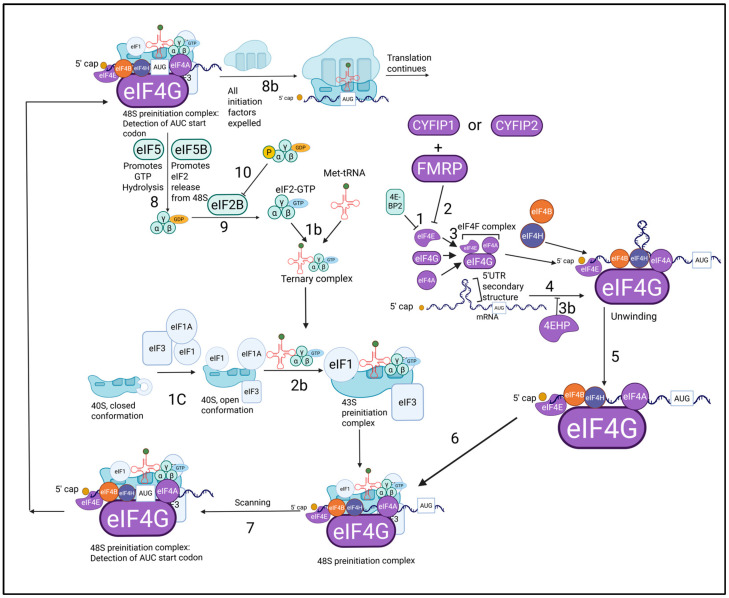
A new comprehensive biochemical flowsheet for protein synthesis initiation and translational control.

After careful analysis and critique of all the literature on protein synthesis initiation and translational control, as seen above, I managed to invent a new comprehensive biochemical flowsheet specifically for protein synthesis initiation and control. What is novel about Figure 2 is that it is a holistic version of all biochemical pathways involved in translational control and protein synthesis initiation, condensed into one flowsheet, whereas other reviews focus primarily on one specific protein signaling pathway, without taking into consideration that the protein or pathway they are studying is part of a much larger, complex process. Figure 2 allows the reader to understand the part and role of each protein in the massive process of protein translation initiation. This figure synthesizes all relevant translational initiation and control information into one biochemical flowsheet. The following is a description of each step of the flowsheet.

Step 1 is the inhibition of eIF4E by 4E-BP2. Step 1b is the binding of Met-tRNA with the eIF2-GTP complex that produces the ternary complex. Step 1c is the binding of eIF1 and eIF1A to the 40S subunit to form the open conformation of the mRNA binding channel, along with the binding of eIF3 to 40S. Step 2 is the inhibition of eIF4E by FMRP and the CYFIP proteins. Step 2b is the binding of the ternary complex to the 40S ribosomal subunit to form the 43S preinitiation complex. Step 3 is the binding of eIF4E and eIF4A to eIF4G to form the eIF4F complex. Step 3b is a different pathway where 4EHP inhibits translation. Step 4 is the cap binding of eIF4E to the 5′ mRNA cap structure. Step 5 is the unwinding of the 5′ untranslated region (UTR) secondary structure by eIF4A, eIF4B, and eIF4H. Step 6 is the binding of the 43S preinitiation complex to the mRNA to form the 48S preinitiation complex. Step 7 is scanning until the UAC codon of the Met-tRNA reaches the AUG initiation codon. Step 8 is the binding of eIF5 to eIF2, causing the subsequent hydrolysis of GTP to GDP, and the binding of eIF5B, causing the expulsion of the eIF2-GDP complex from the 48S preinitiation complex. Step 8b is the expulsion of all other initiation factors from the 48S preinitiation complex, triggered by AUG detection and GTP hydrolysis. Step 9 is the binding of eIF2-GDP to eIF2B and the subsequent exchange of GDP for GTP. Step 10b is the inhibition of eIF2B by excessive binding of eIF2-GDP, caused by eIF2α phosphorylation.

## 4. Neurodegeneration and Oxidative Stress

As described by Hyder et al. [100], brain tissue is especially vulnerable to oxidative stress because of high oxygen consumption due to elevated cortical (brain) energy demands. Moreover, Joffre et al. [101], Carrié et al. [102], Chung et al. [103], Little et al. [104], McNamara et al. [105], and Xiao et al. [106] state that another reason for this vulnerability of brain tissue to high oxidative stress is the high concentration of polyunsaturated fatty acids (PUFAs) found in cortical tissue. As per Sultana et al. [107], these lipids are highly susceptible to lipid peroxidation, a form of oxidative stress. Selley et al. [108], Butterfield et al. [108], Butterfield et al. [109], Dexter et al. [110], Pedersen et al. [111], and Lovell et al. [112] assert that elevated levels of lipid peroxidation products such as 4-hydroxy-2,3-nonenal (HNE) and thiobarbituric acid reactive substances (TBARs) have been found in Alzheimer’s disease, Parkinson’s disease, and amyotrophic lateral sclerosis.

The human body does, however, contain several defense mechanisms against oxidative stress known as antioxidant proteins. According to Pappolla et al. [113], these proteins, such as catalase and superoxide dismutase, have been found to be highly active in association with neurofibrillary tangles (NFTs) and senile amyloid plaques, which are both protein aggregates that are prominent features of Alzheimer’s disease. An important study by Fang et al. [114] also showed that axons are more vulnerable to oxidative stress than soma cell bodies in neurons. As Fang et al. [114] state, oxidative stress disrupts axonal transport, whether it occurs in the axon itself or in the cell body, which leads to axon degeneration.

My analysis led me to conclude that this particular vulnerability to oxidative stress from axons may be due to the lack of proteasomes for the turnover of oxidized proteins. Fusco et al. [115] helped me arrive at this conclusion by explaining how ribosome biogenesis, the production of ribosomes which are necessary for the synthesis of proteasomes, is very limited in the axon compared to the cell body.

Oxidative stress leads to the destruction of neuron cells and neural tissue in several different ways. According to Keller et al. [116] and Pedersen et al. [117], the lipid peroxidation product HNE inhibits glucose and glutamate transport in rat neocortical synaptosomes and motor neurons, respectively. Rat neocortical synaptosomes are in vitro preparations of neuron terminals of the neocortex. The neocortex is, according to Lodato et al. [118], a part of the brain that is involved in higher-order functions such as sensory perception. Mark et al. [119] found that HNE impairs glucose uptake in hippocampal neurons, and hippocampal neurons, as described by Strange et al. [120], are neurons involved in episodic memory. According to Keller et al. [116], HNE disrupts glucose and glutamate transport by impairing the glucose transporter type-3 and glutamate transporter 1 (GLT-1).

Mark et al. [121] state that, in Alzheimer’s disease, amyloid beta-peptide plaques induce peroxidation, which inhibits Na^+^/K^+^-ATPase and Ca^2+^-ATPase, proteins that break down ATP. Poulsen et al. [122] explain that Na^+^/K^+^-ATPase pumps two potassium ions inside the cell and three sodium ions out of the cell. In doing so, the ATPase maintains the balance of ions inside and outside the cell, a concept known as homeostasis. As per Kinoshita et al. [123], the Na^+^/Ca^2+^ exchanger then removes calcium ions from the cell in exchange for three sodium ions, which it brings inside the cell. By inhibiting Na^+^/K^+^-ATPase, the concentration of sodium ions increases inside the cell and decreases outside of it. As a consequence, the Na^+^/Ca^2+^ exchanger can no longer remove calcium ions from the cell, resulting in an increase in intracellular calcium ions. Moreover, as stated by Mark et al. [121], the inhibition of Ca^2+^-ATPase further impairs the cell’s ability to remove calcium ions. This creates an influx of Ca^2+^ within the neuron and, as Hou et al. [124] described, an increase of calcium ion concentration in neurons leads to neuron cell degradation. Hou et al. [124] explain that this is because high calcium ion concentrations open the mitochondria permeability transition pore and release cytochrome c, a protein, which, when released by the mitochondria, activates the caspases, proteolytic enzymes that kill the cell by degrading it.

According to Sorrentino et al. [125], mitochondrial dysfunction in Alzheimer’s, specifically, contributes to the death of the neuron and the synapse. Moreover, as stated by Fang et al. [126], an increase in mitophagy, the elimination of mitochondria that are not functioning properly, has been shown to reverse memory impairment caused by amyloid and tau pathologies. Moreover, Sorrentino et al. [125] and Fang et al. [126] describe that optimal mitochondrial function reduces amyloid β aggregation and inhibits tau hyperphosphorylation, respectively. I will investigate amyloid and tau pathologies in more detail below.

Mitophagy is a subtype of autophagy. As Guo et al. [127] described, autophagy begins when misfolded proteins and organelles are engulfed by a phagophore, created from cellular structures such as the plasma membrane. Guo et al. [127] also explain that once the phagophore fully isolates its target, it becomes an autophagosome. The autophagosome then fuses with a lysosome to form the autolysosome, degrading the target organelle or proteins, a process known as proteolysis.

Autophagy is particularly interesting when studying axon degradation. Lee et al. [128] reported that, in Alzheimer’s disease, an inhibition of lysosomal activity disrupts autophagy and causes pathological axonal swellings, contributing to the axon’s degeneration.

As summarized by Liang et al. [129], mitophagy disruption has been proven to be implicated in neurodegeneration in many different diseases, such as Alzheimer’s disease, Huntington’s disease, Parkinson’s disease (PD), and amyotrophic lateral sclerosis. Mitophagy initiation involves a protein called the phosphatase and tensin homolog-induced kinase 1 (PINK1). Narendra et al. [130] found that proteolysis of PINK1 in healthy mitochondria inhibits this protein’s function. Healthy mitochondria have a specific electric charge difference between their internal and external environments outside of their membrane, known as the membrane potential. This membrane potential triggers the degradation of PINK1. However, this potential is lost in damaged mitochondria, and PINK1 is no longer inhibited. This results in the accumulation of PINK1 in mitochondria, which recruits the parkin protein. As reported by Wang et al. [131], the parkin protein ubiquitinates proteins such as voltage-dependent ion channel-1 (VDAC1). As seen in Harper et al. [132], when interacting with parkin, these ubiquitinated proteins are phosphorylated by PINK1, increasing parkin ubiquitination activity 1000-fold. Parkin is also phosphorylated by PINK1, which increases the strength of binding between ubiquitin and parkin, which promotes further ubiquitin chain assembly. As seen in Wang et al. [131], the ubiquitinated proteins interact with proteins such as p62 which can recruit the phagophore through the interaction between microtubule-associated protein 1A/1B-light chain 3 (LC3) found in the phagophore membrane and p62’s LC3 interacting region (LIR), thereby triggering mitochondrial autophagy (mitophagy).

Mitophagy impairment is mainly caused by (1) specific mitochondrial dysfunctions and/or (2) excessive oxidative stress. In relation to mitochondrial dysfunctions, Coughlin et al. [133] reported that isolated mitochondria in the optic nerves, with an abnormally small surface area and disrupted folds of their inner membrane, were not being efficiently recycled by autophagy, and this preceded axon neurodegeneration. Regarding oxidative stress, Evans et al. [134] reported that oxidative stress renders mitophagy unable to maintain the health of the neuron, leading to neurodegeneration.

The subsequent sections of this paper will focus more on proteasome degradation regarding proteolysis, because 4E-BP2, the key protein of the Unified Theory, has been found, as per Kouloulia et al. [9], to be specifically degraded through the raptor-mediated ubiquitine-proteasome system (UPS) pathway.

In addition to mitophagy, as explained in KaltSchmidt et al. [135], the NF-κB protein can protect neurons from cell death in Alzheimer’s by preventing necroptosis, which is a form of cell death that is very common in Alzheimer’s.

Meffert et al. [136] reported that NF-κB activation depends on synaptic transmission, which goes from axons to neurons. This input causes depolarization, which elevates Ca^2+^ concentration, triggering the activation of Ca^2+^/calmodulin-dependent protein kinase II (CaMKII). CaMKII phosphorylation activates NF-κB. This protein then localizes to the nucleus to cause gene transcription and subsequent protein production. Meffert et al. [136] also demonstrated that NF-κB inactivation led to memory and learning deficits, which are common symptoms of Alzheimer’s.

Bidinosti et al. [8] found that deamidated 4E-BP2 reduces the kinetics of synaptic transmission, making it slower and less efficient. Hernandez-Ochoa et al. [137] showed that slow synaptic transmission and slower depolarization led to low-amplitude calcium changes that were not high enough for proper neuron function.

These findings led me to conclude that slower synaptic transmission caused by deamidated 4E-BP2 inhibits NF-κB activity. This inhibition leads to memory and learning impairment, as well as neuron cell death, which contributes to the pathogenesis of Alzheimer’s.

As per Tamagno et al. [138], hydrogen peroxide (H_2_O_2_) produced by Aβ-peptides leads to the activation of c-Jun aminoterminal kinases (JNKs) and the p38 mitogen-activated protein kinase. This process leads to apoptosis (cell death). P38 and JNKs kill the cell by activating the BAD and BAX proteins, which induce cell death as described in the above section. As stated in Donovan et al. [139], JNK promotes the apoptotic function of the BAD protein by phosphorylating it, and both P38 and JNK, as per Kim et al. [140], promote the apoptotic function of the BAX protein through phosphorylation. Thus, as described above, BAD and BAX proceed to release cytochrome C from the mitochondria, killing the cell. As described in Yamamoto et al. [141] and Inoshita et al. [142], JNK also promotes cell death by suppressing the anti-apoptotic activity of Bcl-2 and Mcl-1 proteins, respectively, through phosphorylation, and, as per Farley et al. [143], p38 also promotes cell death by suppressing the activity of anti-apoptotic proteins like Bcl-2 and Bcl-xl through phosphorylation.

Parkinson’s, on the other hand, as stated in Yang et al. [144], is characterized by a loss of motor function due to the degradation of dopaminergic neurons in the substantia nigra, a region of the brain.

Marsden et al. [145] and Ross et al. [146] described that the crucial role of dopaminergic neurons in motor function is impaired in Pakinson’s patients after roughly 50% of dopaminergic neurons have degraded in the substantia nigra.

As per Cheng et al. [147], in early Parkinson’s disease, when dopaminergic neurons decay, the axons, which are part of synapses, are the first to degrade. Moreover, Bell et al. [148], Gilley et al. [149], Adalbert et al. [150], and Dekosky et al. [151] reported that, in Alzheimer’s disease, synaptic dysfunction and degeneration are found to precede cell body degeneration. Specifically, Dekosky et al. [151] state that synaptic degeneration correlates best with memory loss in Alzheimer’s disease.

Based on my critical analysis, I found that synaptic dysfunction is related to translational control. As seen in the previous section, translational regulation via proteins such as CYFIP1 and CYFIP2 plays a prominent role in neuron development and function, specifically in synapses. Tiwari et al. [88] showed that decreased CYFIP2 translational regulation activity increased synaptic production of amyloid precursor protein (APP) without increasing mRNA expression. Tiwari et al. [88] explain that the decrease in CYFIP2 activity also increased the activity of amyloid precursor protein cleaving enzyme 1 (BACE1). This leads to an increased amount of amyloid beta (Aβ), which polymerizes into pathological Aβ peptides. As described in Marcello et al. [152] and Westmarck et al. [153], amyloid polymerization creates amyloid-beta plaques, which is a process that is highly toxic to synapses and leads to their degeneration and memory loss.

In addition to research on amyloid beta plaques, treatment development for Alzheimer’s has increasingly focused on tau pathology because, as per Tissot et al. [154], the neuropsychiatric symptoms of Alzheimer’s were found to correlate with tau uptake in symptomatic network regions of the brain. Chung et al. [155] state that tau is a microtubule-associated protein that promotes the assembly of microtubules into the cell’s skeleton (the cytoskeleton). As per two published works by Aronov et al. [156,157], Tau mRNA contains an axonal localization cis signal (ALS), which brings Tau mRNA to the axon so that it can be locally translated there. According to Chung et al. [155] and Tzioras et al. [158], this is why Tau is considered primarily an axonal and pre-synaptic protein (the axons form the pre-synapse). Kosik et al. [159] described that tau dysfunction occurs in the neurites, which can be classified as axons or dendrites, that are the neurons’ neuronal projections. Kosik et al. [159] state that tau dysfunction in neurites has been linked to the progression of Alzheimer’s disease. One such dysfunction is hyperphosphorylation. Brion et al. [160], Kenessey et al. [161], and Yu et al. [162] state that tau phosphorylation was found to be significantly higher in fetal tau than in normal adult brain tau. Fetal tau phosphorylation is similar to the phosphorylation found in the pathological tau protein of the paired helical filaments of neurofibrillary tangles. Kenessey et al. [161] reported that phosphorylation overlap between fetal and pathological tau was mainly found near the carboxy-terminal of the protein. However, fetal Tau does not form pathological neurofibrillary tangles.

Kenessey et al. [161] also reported a lack of fetal tau phosphorylation at the Tau-1 epitope and near the amino-terminal compared to pathological tau.

My conclusion is that the lack of phosphorylation at the tau-1 epitope and the amino-terminal in fetal tau is the reason why fetal tau does not form neurofibrillary tangles.

As observed by Yu et al. [162] in embryonic and adult rats, tau phosphorylation is lower in mature brains compared to developing brains due to higher tau phosphatase activity in the adult brain. As reported by Llorens-Martin et al. [163], Kimura et al. [164], Jicha et al. [165], and Liu et al. [166], tau phosphorylation is primarily performed by three kinases: glycogen synthase kinase 3 beta (GSK-3beta) [163], cyclin-dependent kinase 5 [164], and cAMP-dependent protein kinase [165,166]. As reported by Liu et al. [166], tau phosphorylation can be cooperative because prephosphorylation of tau by PKA has been shown to regulate subsequent phosphorylation by CDK and GSK-3beta. As reported by Ando et al. [167], other kinases such as PAR-1/microtubule affinity-regulating kinases (MARKs) also phosphorylate Tau at specific amino acid residues such as serine 262 and serine 356. As reported by Pellarin et al. [168], cyclin-dependent kinases like CDK5 regulate cell cycle progression and have become a popular therapeutic target for cancer treatment using CDK 4/6 inhibitors (Ibrance, Verzenio). As reported by Alonso et al. [169], tau hyperphosphorylation can cause Tau to misfold and form tau “seeds”, which induce tau aggregation, the formation of tau oligomers and neurofibrillary tangles, a highly toxic species that causes synaptic dysfunction. As reported by Hoover et al. [170], hyperphosphorylation also leads to tau mislocalization from the axon to the dendrites and even the dendritic spines (the bulbs at the tip of dendrites). This causes the earliest known synaptic dysfunction in Alzheimer’s by reducing the number of α-amino-3-hydroxy-5-methyl-4-isoxazolepropionic acid (AMPA) receptors on dendritic spines, which are receptors necessary for the transmission of signals and information between neurons. As per Schneider et al. [171], tau hyperphosphorylation at serine 214 is also found in early-onset Alzheimer’s and leads to the disruption of tau-microtubule interaction. Martinez et al. [172] even found that phosphorylation of serine 214 during early onset Alzheimer’s was necessary for the localization and accumulation of toxic tau seed aggregates to the synaptic terminal. This results in the subsequent transsynaptic propagation of the tau seed to other connected neurons as well as other regions of the brain.

Tiwari et al. [88] demonstrated that impairment of translational inhibition proteins like CYFIP2 in early Alzheimer’s disease has led to an increase in serine 214 tau phosphorylation by the CAMKIIα kinase.

Based on these works [88,162,163,164,165,166,167,168,169,170,171,172], since CYFIP2 is a translational control protein, and CYFIP2 inhibition increases Serine 214 phosphorylation in tau, which, in turn, leads to the pathological disruption of tau-microtubule interactions in early Alzheimer’s, I determined that tau pathology is linked to translational control.

Velasquez et al. [173] found that tau-deficient mice had neuronal dysfunction. However, Pickett et al. [174] found that Aβ peptides and tau proteins in Alzheimer’s cooperatively decrease the transcription of genes related to protein function. In the circumstances described by Pickett et al. [174], a decrease in tau levels in mutant mice, where human tau protein production can be turned on and off (reversible expression) using a doxycycline treatment, reversed transcriptional perturbations. Santacruz et al. [175] explain that a Tet-off system controls this reversible expression. Initially discovered by Gossen et al. [176], it is based on a tetracycline-controlled transactivator (tTA) that stimulates tau mRNA transcription. However, tetracycline and its analogs, such as doxycycline, inactivate tTA, which stops the tau gene’s transcription.

Based on my analysis of Pickett et al. [174], an increase in translational control inhibition of tau can stop the progression of Alzheimer’s pathology.

Axonal dysfunction in the early onset of neurodegenerative disease is not specific to Alzheimer’s. As per Wilson et al. [177], synaptic and axonal dysfunction was also found to precede cell death in both amyotrophic lateral sclerosis and multiple sclerosis.

According to Wilson et al. [177], early synaptic and axonal decay also applies to Parkinson’s disease. Stephens et al. [178] describe that early Parkinson’s is caused by soluble α-synuclein proteins, which are primarily enriched in the axon terminal (presynapse). As stated in Maries et al. [179], α-synuclein proteins aggregate to form insoluble fibrillary structures called lewy bodies. Xilouri et al. [180] reported that alpha-synuclein accumulation is likely due to a decline in activity by the ubiquitine-proteasome system (UPS) and the autophagy-lysosome pathway (ALP) due to aging, resulting in the accumulation of alpha-synuclein. As seen in Spano et al. [181], mitochondrial dysfunction has also been shown to contribute to the formation and accumulation of lewy bodies. As explained in Tain et al. [182], the mutations of the PINK1 and Parkin genes create mitochondrial dysfunction, which subsequently leads to dopaminergic neuron cell death. Tain et al. [182] explain that dopaminergic neuron cell death due to PINK1 and parkin mutant pathology can be prevented by 4E-BP activation.

In addition to Xilouri et al. [180], Hoshino et al. [183] found that age-related UPS decline in function leads to the increased production of proteins encoded in genes such as N-myc downstream-regulated gene 1 (NDRG1), which cause neurodegeneration upon overproduction. Based on my analysis of this information, pathological protein aggregation in neurodegeneration in ageing patients is a direct consequence of a decline in activity by protein degradation systems, such as the ubiquitine-proteasome system.

Zhang et al. [184] developed a drug called Synucleozid 1.0 to decrease α-synuclein levels by targeting α-synuclein mRNA (SNCA mRNA). As per Tong et al. [185], SNCA mRNA, which undergoes cap-dependent translation, contains an iron-responsive element (IRE) in its 5′ untranslated region. Tong et al. [185] explain that iron regulatory proteins (IRPs) bind the IRE at low iron concentrations, repressing α-synuclein translation, whereas IRPs bind iron instead of the IRE at high iron concentrations, allowing α-synuclein translation to progress. Synucleozid 1.0 mimics IRP activity and binds to IRE, prohibiting α-synuclein translation. However, Tong et al. [185] state that Synucleozid 1.0 could not be used as an effective treatment for Parkinson’s, because it could not effectively penetrate the central nervous system.

In addition to lewy bodies, Shukla et al. [186] state that, as seen in the previous paragraphs of this review, oxidative stress has also been shown to contribute directly to the neurodegeneration of dopaminergic neurons in Parkinson’s.

I observed a clear pattern throughout this section: overexpression of certain proteins whose translation is controlled by 4E-BPs leads to neurodegeneration. In fact, Creus-Muncunill et al. [187] found that 4E-BP inactivation results in protein overexpression, which leads to neurodegeneration in diseases such as Huntington’s disease. As per Li et al. [188], Huntington’s pathology also originates in neuronal projections, mainly axons, just like Parkinson’s and Alzheimer’s, as previously reported in this paper.

My critical analysis and review of the relevant scientific literature suggest that protein synthesis dysfunction, translational control dysfunction, and protein degradation dysfunction work in synergy to progress neurodegeneration. The following section will discuss in detail how my discovery that 4E-BP2 deamidation is specific to neuronal projections, such as axons, opens the door to effective treatments against this synergy of dysfunction.

## 5. My Discovery

My work [10] discovered the fundamental mechanism behind neuron-specific 4E-BP2 deamidation. My experimental results confirmed that 4E-BP2 deamidation mainly occurs in the neuron’s proteasome-poor axons. I concluded [10] that this was because this proteasome-poor environment, unique to neurons, allowed proteins to have longer half-lives than the half-life of deamidation.

Several studies have provided isolated facts that prove my theory:

(1) Kouloulia et al. [9] showed that proteasomal inhibition increases 4E-BP2 deamidation.
(2) Sun et al. [189] reported that both axons and dendrites are proteasome-poor environments, through immunolabelling of rat cortical neurons and immunofluorescence staining of the mouse stratum pyramidale in the hippocampus.

My paper was the first to establish that neurites, the neuronal projections of the soma, mainly axons, are the reasons why deamidation happens only in neurons.

Sun et al. [189] confirmed that a proteasome-poor environment is also present in dendrites, which are smaller neuronal projections, like axons.

As a consequence, I concluded that 4E-BP2 deamidation must also occur in this other type of neuronal projection. However, deamidation occurs mainly in axons, because axons are by far the largest neurites.

I had considered isolating the stratum pyramidale (mainly cell bodies) and stratum radiatum (mainly dendrites) to compare deamidation rates using immunoblotting, but I chose not to proceed with this experiment, because, as Szabo et al. [190], Ellender et al. [191], and Tyzio et al. [192] have described, pyramidal neuron cells from the stratum pyramidale are heavily innervated by GABAergic “basket cell” neurons, which are inhibitory neurons, and are innervated by many other types of neurons as well. As a result, the high density of neuronal projections around the cell bodies inside the hippocampus is not separable, and dissection of the stratum pyramidale would not give a pure enough sample of cell bodies for immunoblotting.

I postulated that 4E-BP2 neural tissue-specific deamidation was due to axons and their unique properties [10]. In order to validate this, I dissected the optic nerve and the retina, because, according to London et al. [193], Zhao et al. [194] and Butt et al. [195], they were extensions of the diencephalon and therefore the brain and central nervous system.

Subsequently, I dissected the sciatic nerve and its respective dorsal root ganglia to verify my results in the peripheral nervous system, because, as seen in Prakash et al. [196] and Murakamai et al. [197], the sciatic nerve was isolatable via dissection and is the longest nerve in humans and mice, respectively.

**Figure 3:**
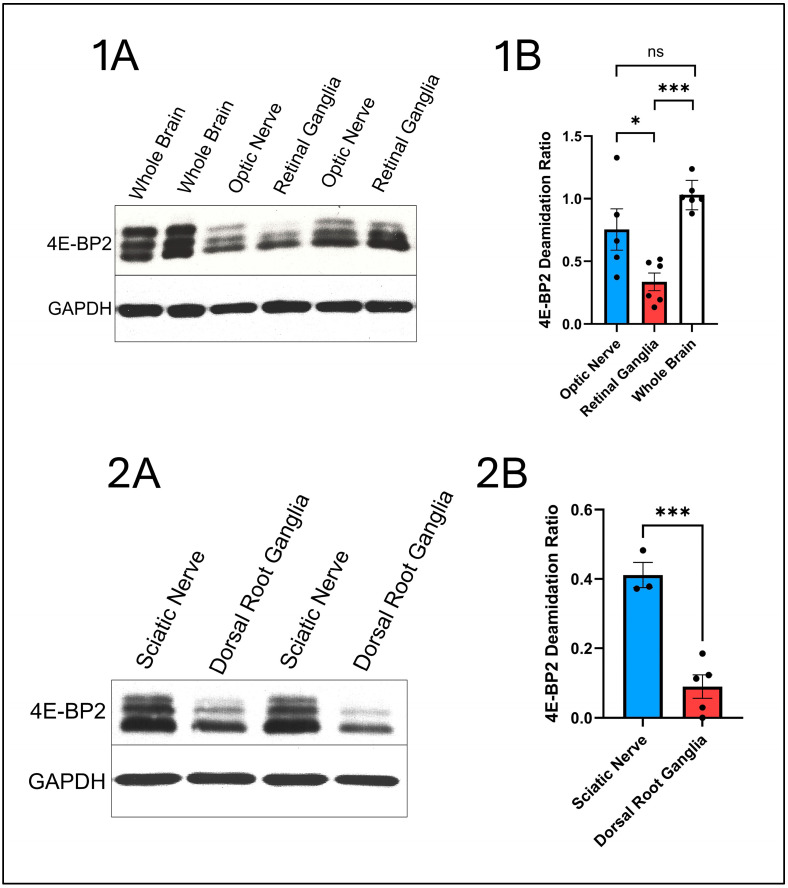
Immunoblotting (**1A**,**2A**) and statistical analysis results (**1B**,**2B**) of 4E-BP2 deamidation in the whole brain (**1A**), the optic nerve (**1A**), the retinal ganglia (**1A**), the sciatic nerve (**2A**) and the dorsal root ganglia (**2B**), reproduced in a different format from my previously published paper [10]. In this figure, “ns” is a non-significant *p*-value, where *p* > 0.05, “*” is a significant *p*-value, where *p* < 0.05, and “***” is a *p*-value of higher significance, where *p* < 0.001.

The results from my 2024 paper are shown in Figure 3.

The next step in my research was to compare the effect of myelination on deamidation. As Williams et al. [198] described, the central nervous system is primarily myelinated, whereas Schmalbruch et al. [199] showed that the nerves of the peripheral nervous system, such as the sciatic nerve are mostly unmyelinated. In fact, Schmalbruch et al. demonstrated that 48% of sciatic nerve axons are unmyelinated sensory axons and 23% are unmyelinated sympathetic axons, which means a total of 71% of sciatic nerve axons are unmyelinated.

I discovered that 4E-BP2 deamidation was found to be significantly higher in myelinated axons [10].

The next step in my work was the interpretation of my data. The key to deciphering the neural tissue-specific deamidation mechanism was my review of previous literature regarding superoxide dismutase (SOD1) and its half-life in various tissues.

As per Hoffman et al. [200], SOD1 has been found to have a half-life of only 100 hours in kidney cells but, as demonstrated in Borchelt et al. [201], SOD1 has a half-life of over one year in axons, where it undergoes axonal transport via the slow component b in motor neurons. As seen in Black et al. [202], the “slow component b” was found to transport proteins at a rate of 2 mm a day, and transport distances across motor neurons can exceed one meter in length. Sleigh et al. [203] also describe travel times for the protein as exceeding one year.

Therefore, I concluded that SOD1 proteins had a half-life exceeding one year in order to be able to survive this very long travel time.

Shi et al. [204] previously hypothesized that this increase in half-life was due to axonal transport. This hypothesis, however, did not stand up to the scrutiny of my literature review for the following reasons:

1. My experimental results found no evidence of deamidation rates increasing as axon length increased. On the contrary, the average deamidation ratio was higher in the shorter optic nerve axons (ratio = 0.7539) than in the longer sciatic nerve axons (0.4112).
2. There is no long-term transport conducted within erythrocytes (red blood cells). However, as per Rolfs et al. [205], proteins specific to erythrocytes, such as hemoglobin, exhibit significantly longer half-lives, especially compared to proteins found in plasma. According to Smith et al. [206], plasma proteins undergo extracellular transport, yet I found no evidence that the transport itself significantly increases the transported proteins’ half-life.

Robinson et al. [207] postulated that every asparagine has one specific deamidation half-life that is determined by the peptide sequence surrounding it, as well as the secondary and tertiary structures of the protein. This, as well as Hoffman et al. [200] and Borchelt et al. [201] cited above, helped me conclude that 4E-BP2 deamidation is caused by axons, which increase the half-life of the protein.

The next step in my analysis was to determine what properties specific to the axon made deamidation possible inside it based on my research results.

The results in my statistical analysis of the impact of myelination on 4E-BP2 deamidation helped me identify this deamidation-causing property.

As seen in Figure 3, the difference in deamidation rates is much more significant between the sciatic nerve and the dorsal root ganglia (*p* < 0.001) than is the difference between the retinal ganglia and the optic nerve (*p* = 0.03). After reviewing the scientific literature, I concluded that this is certainly due to the level of purity in cell body samples. I stripped the retina of the vast majority of its axons by separating it from the optic nerve, and the retina samples were much more enriched in cell bodies compared to the optic nerve, which is made up of pure axons and no cell bodies. While it is true that the optic nerve fiber contains, in small amounts, types of tissue other than axons, such as oligodendrocytes, immune cells, and blood vessels, Bidinosti et al. [8] and Kouloulia et al. [9] already confirmed that 4E-BP2 is found mainly in axons and that 4E-BP2 deamidation is neuron-specific. Therefore, the 4E-BP2 deamidation detected in my nerve samples is coming from the axons. 

However, even though the vast majority of retina axons and retina axon mass are found in the optic nerve, some axons remain in the retina after optic nerve removal. These axons are found in the synapses between photoreceptors and bipolar cells and between the bipolar cells and the retinal ganglia. Xiao et al. [208] describe how photoreceptors and bipolar cells transmit information to the retinal ganglia through these synapses. The dorsal root ganglia (DRG), on the other hand, as reported in Krames et al. [209], are entirely devoid of synaptic input from other neurons because no synapses are formed inside the DRG, making DRG even poorer in axons than my retina samples, and, as predicted by my theory, DRG are the samples that demonstrated the poorest amount of deamidation.

This confirms my theory: the purer the sample in cell bodies, the more significant the difference in deamidation ratios between cell body samples and deamidation-enriched axon samples.

As previously mentioned in Rolfs et al. [205], erythrocyte proteins exhibit significantly longer protein half-lives. In order to decipher the mechanism behind this longer protein half-life, I investigated and discovered what made erythrocyte protein degradation unique compared to protein degradation in other cells. The uniqueness is that, according to Neelam et al. [210], 26S proteasomes, which degrade ubiquitinated proteins (proteins marked for degradation), are found in very poor abundance in erythrocytes. Neelam et al. [210] also found that the degradation activity of 26S proteasomes is insignificant in erythrocytes. As seen in Rosenzweig et al. [211], 26S proteasomes are made up of two subunits: the 20S core particle, which performs proteolytic activity (protein degradation), and the 19S regulatory particle, which detects the ubiquitin signal and unfolds the protein. Sae-Lee et al. [212] described that only 6% of 20S proteolytic core particle subunits form 26S proteasomes in the erythrocyte. This means a very low abundance of 26S proteasomes exists in erythrocytes, as confirmed by Neelam et al. [210]. While the 20S subunit is present in the erythrocyte, it can only degrade misfolded proteins, because 20S does not contain the 19S regulatory particle necessary for detecting the ubiquitin tag and unfolding the protein. As seen in Sahu et al. [213], only the 26S proteasome can correctly recognize folded, functional, ubiquitinated proteins and degrade them.

Kouloulia et al. [9] showed that wild-type 4E-BP2 and deamidated 4E-BP2 were found to be degraded via the ubiquitin-proteasome system (UPS). Deamidation increased ubiquitination and proteasomal degradation of 4E-BP2.

Based on my analysis of the above-mentioned literature [8,9,189,190,191,192,193,194,195,196,197,198,199,200,201,202,203], I concluded that deamidated 4E-BP2 will accumulate in regions where the 26S proteasome is absent, because 4E-BP2 will not be degraded. I also concluded that these regions are the axons, which were found by Sun et al. [189] to be poor in 26S proteasomes due to the 20S subunit being significantly more abundant in the cell body, even though axons contain 90% of the proteome. Axons are poor in 26S proteasomes because, as per Fusco et al. [115], ribosome biogenesis is mainly performed in the cell body.

Furthermore, I [10] postulated that the major difference in 4E-BP2 deamidation between myelinated and unmyelinated neurons was because of a difference in proteasome abundance between these two tissues. The reasoning for this postulation is given below:

When looking at proteasome abundance in the central nervous system (CNS), Minagar et al. [214], found that the CNS had a significant increase (*p* = 0.01) in proteasome quantity and proteasome enzymatic activity in patients with Multiple Sclerosis (MS), and they inhibited the increase in proteasome enzymatic activity using IFN-beta-1b. I deduced that this fluctuation in proteasome enzymatic activity and quantity found by Minagar et al. [214] was specific to neuronal proteasomes because, according to Kieseier et al. [215], IFN-beta-1b targets neurons specifically by reducing neuron inflammation. As stated in Woo et al. [216], multiple sclerosis is an autoimmune disease that causes demyelination. I discovered that Minagar et al. [214], without intending to study proteasome abundance in unmyelinated neurons, found that proteasome expression increased in neurons after myelin was removed.

Moreover, and most importantly, I reasoned that unmyelinated neurons had higher proteasome production because, as seen in Neishabouri et al. [217], they consume 70 times more ATP and synthesize much more protein than myelinated neurons, such as ion channel proteins. In support of my reasoning, Deng et al. [218] described that protein synthesis is the driving force behind higher ATP consumption, and Zhang et al. [219] described that cells with increased protein synthesis have an increased protein degradation capacity because of higher proteasome abundance.

This reasoning, based on the scientific literature, confirms my postulation on unmyelinated proteasomes mentioned above.

My findings [10] led to the Davis-Joseph principle, stated below:

“Due to their proteasome-poor environment, axons increase the protein half-life, which becomes more significant than the deamidation half-life of asparagines, creating neuron-specific deamidation.”

Since dendrites have also been found to be poor in proteasome abundance, I conclude that deamidation is also found in dendrites significantly.

Therefore, in the spirit of clarity, a generalization can be added to my principle:

“Due to their proteasome-poor environment, neuronal projections, consisting mainly of axons, increase the protein half-life to a level greater than the deamidation half-life of asparagines, creating neuron-specific deamidation.”

My principle also explains the evolutionary advantage of having 4E-BP2 primarily in the neurons: 4E-BP2’s ability to deamidate increases its affinity for raptor, which induces its ubiquitination and subsequent degradation, a process that is necessary in a proteasome-poor environment where protein degradation is less efficient.

This generalization is supported by the results of my previous work [10], where, as seen in Figure 3, the difference in deamidation was far greater between the DRG and its axons (*p* < 0.001) than between the retinal ganglia and its axons (*p* = 0.03), because the retinal ganglia samples contained small amounts of synapses made up of axons and dendrites, whereas the DRG contained none.

As validated in my previous work [10] and explained in this critical review, deamidation is neurite-specific, mainly occurring in the axons because axons are much larger than dendrites.

The following literature corroborates my principle:

(1) The findings of Ritchie Truong [220] provide further evidence in support of my principle. Truong [220] concluded that 4E-BP2 deamidation was significant in skeletal muscle tissue but not in the heart muscle. As per Ruff et al. [221], skeletal muscle contraction depends on innervation via the axons found in neuromuscular junctions.
(2) Based on Ruff et al. [221], my analysis shows that axon terminals are abundant in skeletal muscle tissue, which explains the presence of significant 4E-BP2 deamidation in muscle tissue. As described by His et al. [222] and Difrancesco et al. [223], cardiac muscle propulsion, however, is independent from motor axon input from the nervous system, with specialized myocardium cells being able to spontaneously generate action potentials.

Thanks to my critical analysis review, 4E-BP2 deamidation is established as a therapeutic target for neurodegeneration.

This work paves the way for treating multiple neurodegenerative diseases with the same unified approach based on the therapeutic control of 4E-BP2 deamidation.

This important conclusion on the unified approach of 4E-BP2 therapeutic control is based on my findings in my previous research paper [10] as well as my critical analysis of the relevant scientific literature in the above sections. Specific sections of my theory are also corroborated by the following publications that were already cited above in this paper and repeated together here, side-by-side for consistency, in addition to an important point provided by Guo et al. [224] regarding oxidative stress:

(1) Robinson et al. [2] described that deamidation leads to the programmed dysfunction of proteins
(2) Bidinosti et al. [8] discovered that deamidation occurs in 4E-BP2, which functions as a translational inhibitor. He also reported that 4E-BP2 deamidation reduces the speed and efficiency of 4E-BP2 deamidation.
(3) Tiwari et al. [88] demonstrated that translational control dysregulation from 4E-BP proteins like CYFIP2 causes the early development of neurodegenerative diseases such as Alzheimer’s.
(4) Joseph [10] discovered that 4E-BP2 deamidation is caused by the proteasome-poor environment in neuronal projections, consisting mainly of axons.
(5) Cheng et al. [147] and Dekosky et al. [151] reported that neurodegeneration occurs first in the axons and synapses in Alzheimer’s, Parkinson’s and other neurodegenerative diseases.
(6) Xilouri et al. [180] and Hoshino et al. [183] stated that a decrease in proteasome activity in ageing patients has been linked to the progress of neurodegenerative diseases due to the accumulation of pathological proteins.
(7) Kouloulia et al. [9] found that deamidated 4E-BP2 overexpression is caused by proteasome inhibition and has been shown to decrease the expression of many genes implicated in cerebral cortex development and mitochondrial function, and that deamidated 4E-BP2 also decreases the activity of NF-κB in neurons.
(8) Sorrentino et al. [125] asserted that mitochondrial dysfunction has been found to contribute to neuron cell death in Alzheimer’s and Parkinson’s
(9) Guo et al. [224] explained that mitochondrial dysfunction causes oxidative stress.
(10) Fang et al. [114] showed that axons are most vulnerable to oxidative stress
(11) Sorrentino et al. [125] and Fang et al. [126] stated that the reduction of dysfunctional mitochondria via mitophagy in neurons reduces pathological Aβ aggregation, inhibits pathological tau hyperphosphorylation and reverses memory impairment caused by tau and amyloid pathology.
(12) Tain et al. [182] described that 4E-BPs inhibit PINK1 and Parkin protein mutants responsible for mitophagy disruption and dopaminergic neuron degradation in Alzheimer’s.
(13) Kaltschmidt et al. [135] postulated that NF-κB protects neurons from necroptosis.
(14) Meffert et al. [136] reported that NF-κB activation depends on synaptic transmission, and that NF-κB inactivation leads to learning and memory impairment.
(15) Tiwari et al. [88], Li et al. [72] and Tong et al. [185] demonstrated that amyloid precursor protein and tau (both involved in Alzheimer’s pathology) as well as alpha-synuclein (involved in Parkinson’s pathology) all undergo cap-dependant protein translation involving eIF4E that is inhibited by 4E-BPs.
(16) Creus-Muncunill et al. [187] reported that excessive protein synthesis after 4E-BP inhibition results in neurodegeneration in diseases such as Huntington’s disease.

My Unified Theory of neurodegeneration pathogenesis, based on axon deamidation, originates from my discovery of the fundamental neurobiological mechanism behind neuron-specific 4E-BP2 deamidation in my previous paper [10] as well as my critical analysis of the scientific literature.

It is described as follows:

“4E-BP2 deamidation rates in the neuronal projections, consisting mainly of axons, control the occurrence and progression of neurodegenerative diseases, including Alzheimer’s and Parkinson’s, and establish a unified regulatory system between four different biochemical processes: deamidation, translational control, neurodegeneration, and oxidative stress.”

**Figure 4:**
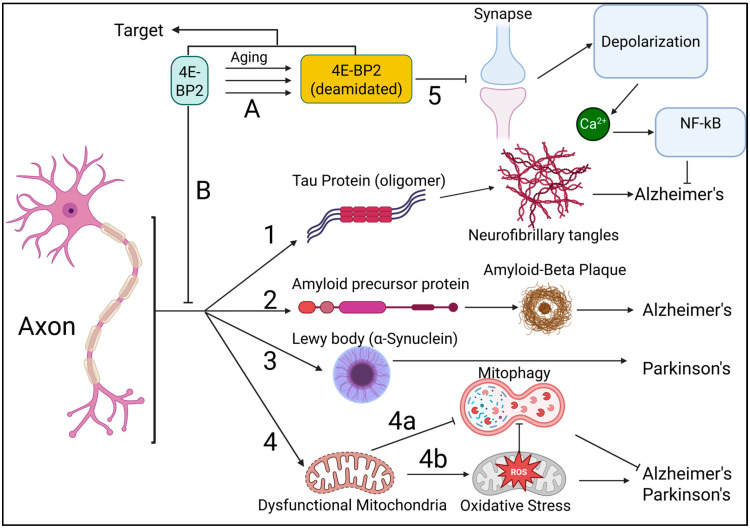
The Unified Theory of Neurodegeneration Pathogenesis, which is based on axon deamidation, establishes the link between the four fields of deamidation (Step A), translational control (Step B), neurodegenerative diseases (Options 1 to 4), and oxidative stress (Option 4).

My Unified Theory is expressed graphically in Figure 4:

In ageing patients, it is observed that there is a decrease in the activity of the ubiquitine-proteasome protein degradation system below healthy rates. As shown in Figure 4, this causes 4E-BP2 deamidation to happen above the normal rates in axons and synapses, the segments of the neuron most vulnerable to neurodegeneration (Step A in Figure 4). As a result, there is less remaining undeamidated 4E-BP2 available to inhibit the overproduction of pathological precursors of neurodegenerative diseases (Step B), namely tau (Option 1), Aβ (Option 2), and α-synuclein (Option 3), as well as mitochondrial dysfunction (Option 4), which inhibits mitophagy (4a) and produces mitophagy dysfunction- inducing reactive oxygen species (ROS, 4b) that cause oxidative stress, which also inhibits mitophagy. All of these cause the occurrence and progress of neurodegenerative diseases such as Alzheimer’s and Parkinson’s. 4E-BP2 deamidation overproduction has interconnected negative consequences, because an increase in 4E-BP2 deamidation (step A) causes a decrease in synaptic transmission-induced NF-kB activity, which, in turn, decreases the chances of survival of the neuron under stress in Alzheimer’s (Option 5).

Therefore, 4E-BP2 deamidation inhibition can prevent:

(1) Aβ aggregation and synapse loss in Alzheimer’s.
(2) The formation of neurofibrillary tangles and resulting memory loss in Alzheimer’s
(3) The formation of Lewy bodies in Parkinson’s
(4) Dysfunctional mitochondria accumulation, which causes oxidative stress in Alzheimer’s and Parkinson’s
(5) Early neurodegeneration in axons and synapses
(6) A decrease in synaptic transmission induced NF-κB activity, which results in neuron death

4E-BP2 deamidation is, therefore, a therapeutic target for local neuron translation in the regions that are most vulnerable to neurodegeneration and oxidative stress, axons and synapses, as per my discovery on the role of axons in 4E-BP2 deamidation.

This is shown in Figure 4 as a 4E-BP2 deamidation “Target”. The target’s purpose is to control 4E-BP2 deamidation rates in order to keep them at the optimum level for healthy neurons. In the case of 4E-BP2 deamidation overproduction, inhibition will increase the abundance of undeamidated 4E-BP2 in the neuron and inhibit pathological accumulation of amyloid plaques, tau, α-synuclein, and dysfunctional mitochondria. 4E-BP2 deamidation inhibition, in this case, can also increase NF-κB activity, which protects the neuron from necroptosis (death).

The specific disease that occurs in patients will depend on the patient’s genetic background and exposure to certain environmental factors.

## 6. Conclusions

In this paper, I developed the Unified Theory of neurodegeneration pathogenesis based on axon deamidation, which is defined as follows:

4E-BP2 deamidation rates in the neuronal projections, consisting mainly of axons, control the occurrence and progression of neurodegenerative diseases, including Alzheimer’s and Parkinson’s, and establish a unified regulatory system which applies to four different biochemical processes: (1) deamidation, (2) translational control, (3) neurodegeneration, and (4) oxidative stress.

I developed this theory by conducting a thorough and critical review of 224 scientific publications regarding deamidation, translational control in protein synthesis initiation, neurodegeneration, oxidative stress, and by applying my discovery [10] of the fundamental neurobiological mechanism behind neuron-specific 4E-BP2 deamidation to practical applications in medicine.

Based on my newly developed theory and my critical review of the scientific literature, I also designed three biochemical flowsheets of (1) in-vivo deamidation, (2) protein synthesis initiation and translational control, and (3) 4E-BP2 deamidation as a control system of the four biochemical processes studied in detail in my paper.

I also generalized the principle of my discovery [10] from the previous paper, where deamidation was found to happen in axons because of their proteasome-poor environment, to a generalized one that includes neuronal projections like dendrites, which were found, based on my literature review and my previous article’s results [10], also to be proteasome-poor. The new generalized principle is written below:

“Due to their proteasome-poor environment, neuronal projections, consisting mainly of axons, increase the protein half-life to a level greater than the deamidation half-life of asparagines, creating neuron-specific deamidation.”

The Unified Theory of Neurodegeneration Pathogenesis, based on axon deamidation developed in this work, paves the way to controlling the occurrence and progression of neurodegenerative diseases such as Alzheimer’s and Parkinson’s through a unique, neuron-specific regulatory system that is 4E-BP2 deamidation, caused by the proteasome-poor environment in neuronal projections, consisting mainly of axons.

As such, 4E-BP2 deamidation control is the crucial therapeutic target for neurodegenerative diseases, especially for axons and synapses, which are most vulnerable to neurodegeneration and oxidative stress, where 4E-BP2 deamidation mainly occurs.

## Data Availability

The original contributions presented in this study are included in the article. Further inquiries can be directed to the corresponding author.
